# Supplementary material for: The genetic and evolutionary basis of gene expression variation in East Africans
Source: Genome Biol. 2023 Feb 24;24:35. doi: 10.1186/s13059-023-02874-4 (PMC9951478; doi:10.1186/s13059-023-02874-4)
Supplement: Supplementary file 1 — Additional file 1: Fig S1. Principal Component Analysis of East African and 1000 Genome Project. Fig S2. ADMIXTURE analysis across K values 2-12. Fig S3. Genomic context of tQTLs. Fig S4. π1 of eQTL p-values of SNP-gene pairs ascertained as sQTLs. Fig S5. π1 between sQTLs and eQTLs across gene length deciles. Fig S6. π1 value of ascertained eQTLs and sQTLs in GTEx. Fig S7. Mapping statistics from STAR. Fig S8. Frequency and LD differences between African samples and 1000 Genomes EURpopulations. Fig S9. tQTL effect size vs MAF. Fig S10. Fraction of FST outliers among eQTLs and sQTLs compared with matched background. Fig S11. Population-specific FST outliers. Fig. S12. Global frequencies of SNPs associated with pigmentation variation and TMEM216 expression and splicing. Fig S13. Colocalization of Mursi PBS and d-statistics with TMEM216 eQTLs. Fig S14. Colocalization of Mursi PBS and d-statistics with pigmentation GWAS. Fig S15. eQTL associations for TMEM216 across populations. Fig S16. ‘LocusCompare’ plots of African pigmentation GWAS and GTEx v8 eQTLs. Fig S17. ‘LocusCompare’ plots of African pigmentation GWAS and GTEx v8 sQTLs. [file 13059_2023_2874_MOESM1_ESM.pdf]

Supplemental Figures

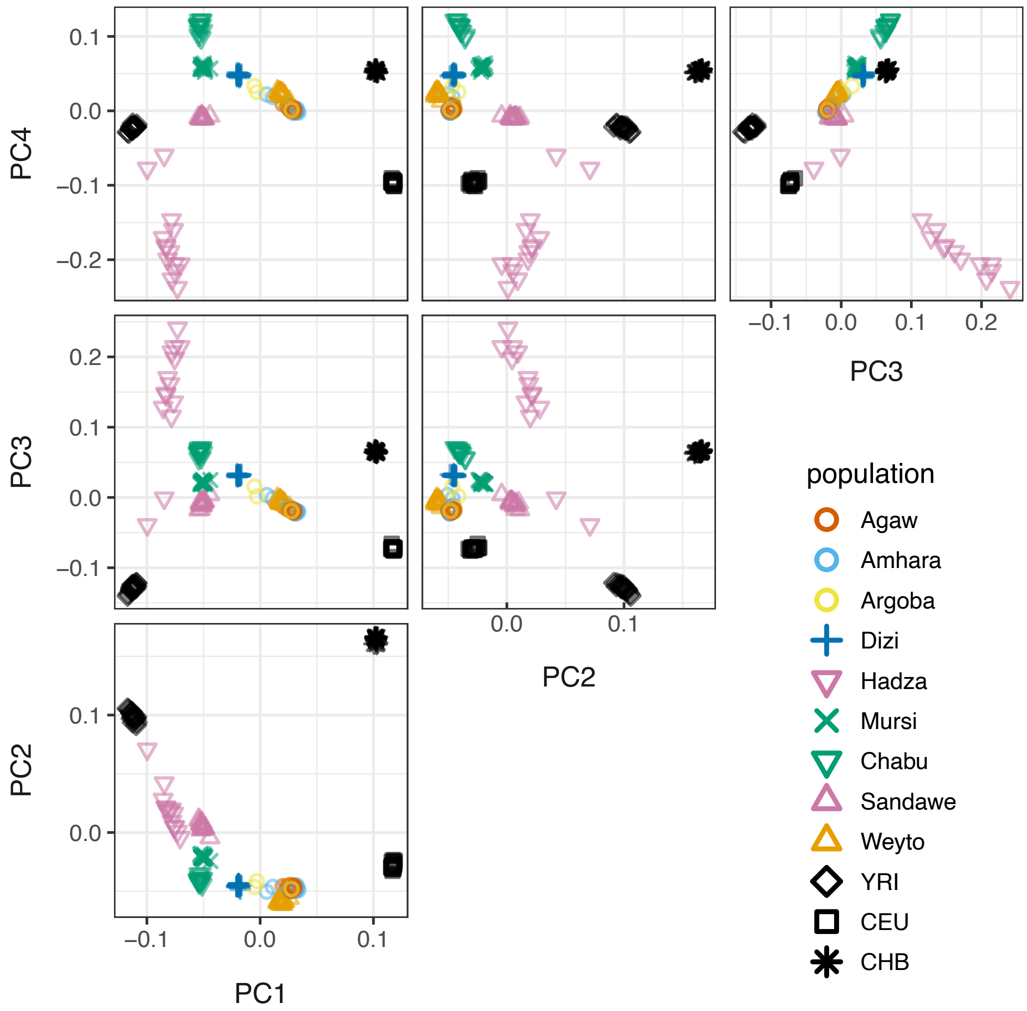

Fig. S1. Principal Component Analysis of East African and 1000 Genome Project populations

Principal Component Analysis was performed on a merged and LD-pruned genotype dataset consisting of 145 East African individuals (filtered for relatedness) and 20 individuals each from the YRI, CEU, and CHB populations (methods). Pairwise plots of principal components (PCs) 1-4 are shown, colored by population label.

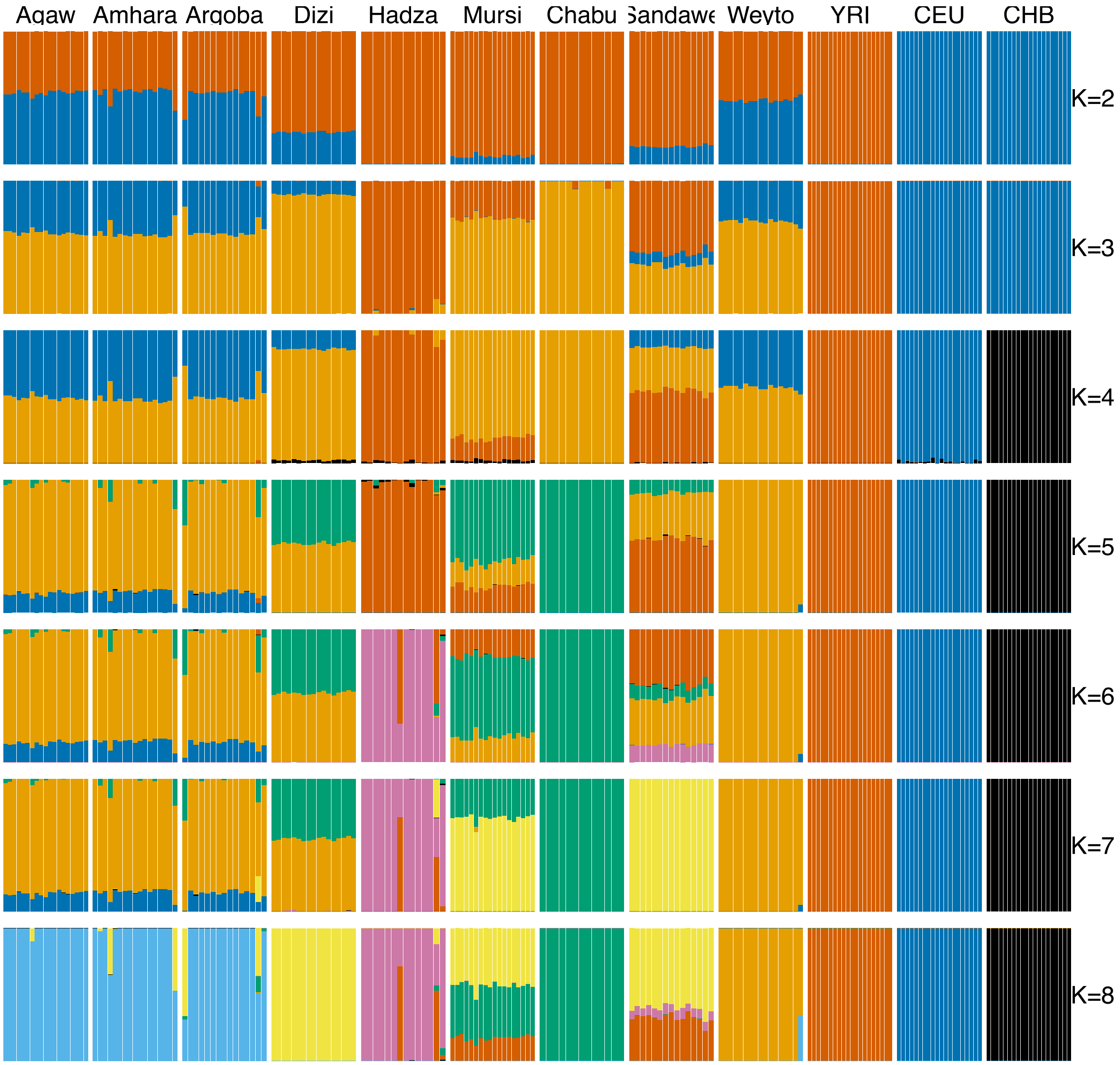

Fig. S2. ADMIXTURE analysis across K values 2-12

ADMIXTURE analysis was performed on the merged and LD-pruned dataset used for PCA, and run for 2-8 clusters (methods). K=2 shows clear separation between Africans and non-Africans, with evidence of non-African admixture among several populations.

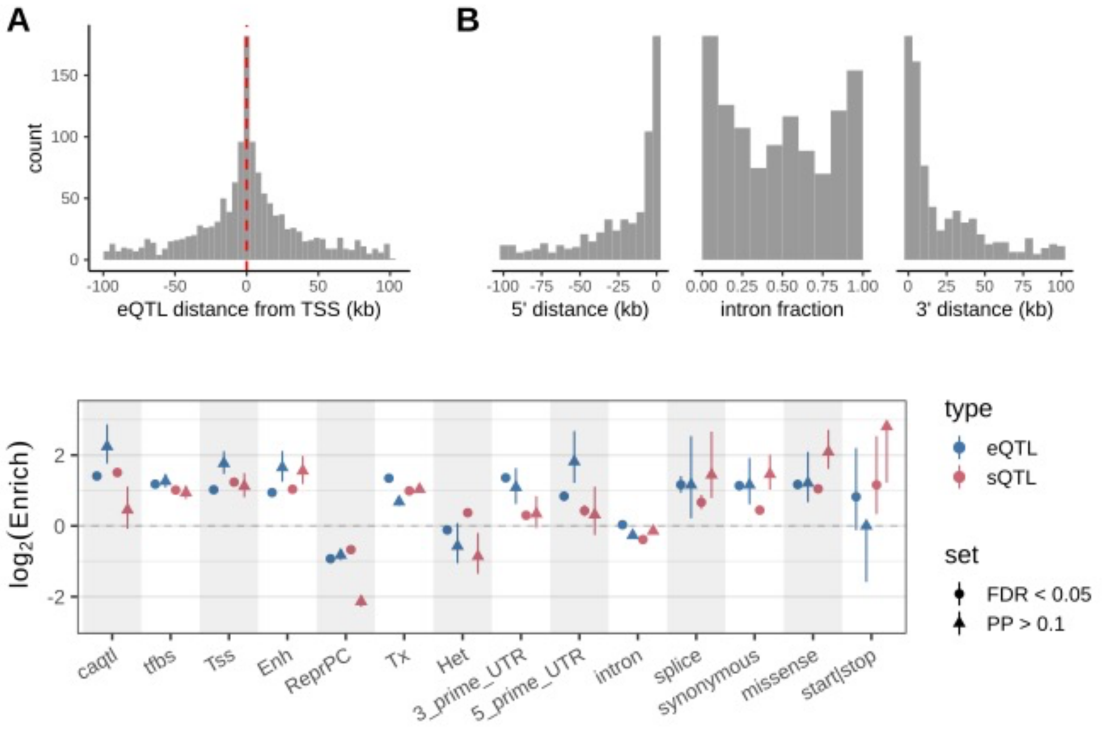

Fig. S3. Genomic context of tQTLs

A) Enrichment of top eQTLs near the transcription start site (TSS) of their target gene. **B)** Enrichment of top sQTLs near the intron boundary of their target intron. Densities of sQTLs are separated depending on whether they're upstream of the target intron (5' distance), within the intron (intron fraction), or downstream of the intron (3' distance). **C)** Enrichment of tQTLs across functional categories, stratified by FDR significance and posterior probability (PP) of being causal. Categories include chromatin accessibility QTLs (caQTL) in LCLs from Tehranchi *et al.* [1]; transcription factor binding sites (TFBS) for 140 transcription factors in GM12878 LCLs [2]; transcription start sites (TSS), enhancers (Enh), Polycomb-repressed chromatin (ReprPC), transcribed (Tx), and heterochromatin (Het) annotations from ChromHMM in GM12878 LCLs [2]; and 3' UTR, 5' UTR, intron, splice site, synonymous, missense, and start gain/loss or stop gain/loss annotations from Variant Effect Predictor (VEP) [3].

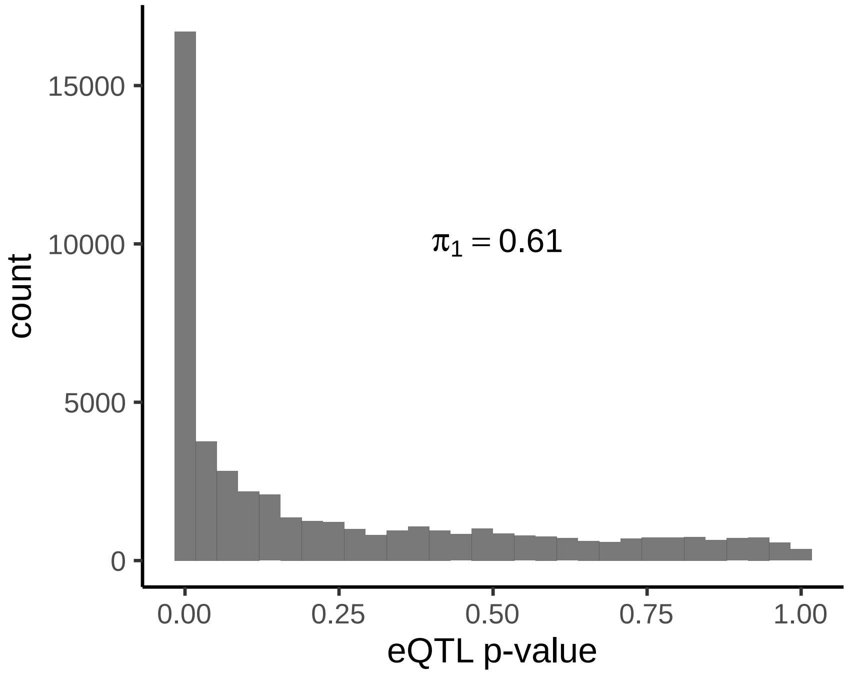

Fig. S4. π_1 of eQTL p-values of SNP-gene pairs ascertained as sQTLs

SNP-intron pairs that meet FDR-significance are merged at the gene level to generate unique SNP-gene pairs. The π_1 is then estimated from the eQTL scan p-values of these SNP-gene pairs to approximate the number of true positives, and thus fraction of sQTLs that are also eQTLs.

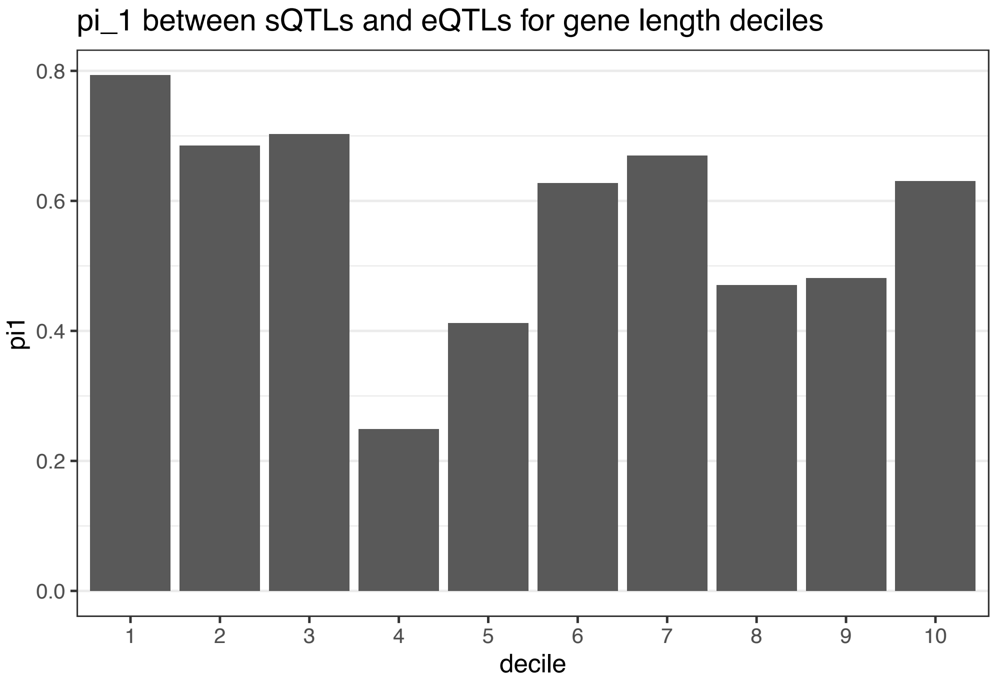

Fig. S5. π_1 between sQTLs and eQTLs across gene length deciles

To test whether RNA-seq artifacts related to gene length were driving the replication between sQTLs and eQTLs, we performed estimation of π_1 for gene binned into deciles by gene length. We find that the shortest genes show the strongest enrichment, but all bins show appreciable replication.

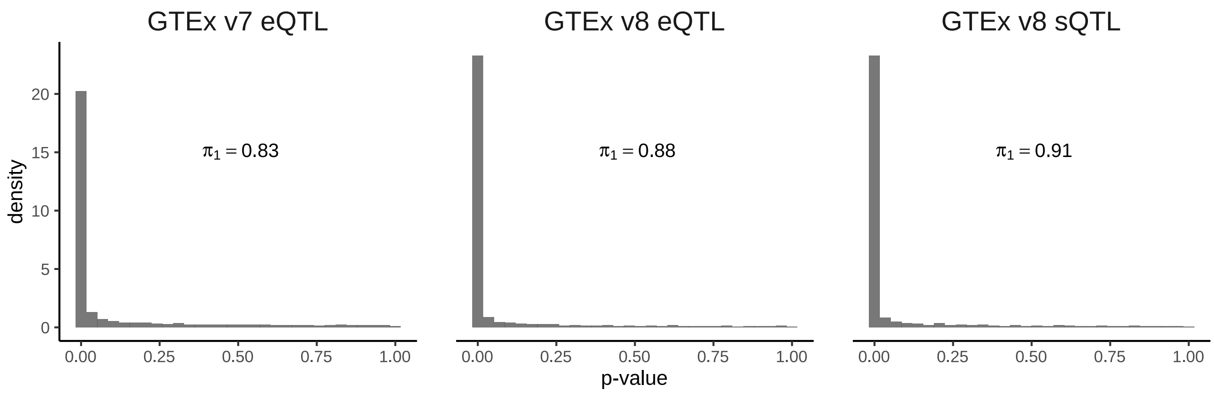

Fig. S6. π_1 value of ascertained eQTLs and sQTLs in GTEx

P-values of SNP-gene (eQTL) or SNP-intron (sQTL) pairs that meet FDR-significance in our African cohort are extracted from GTEx (v7 and v8 for eQTLs, v8 for sQTLs). π_1 is then estimated from these p-values.

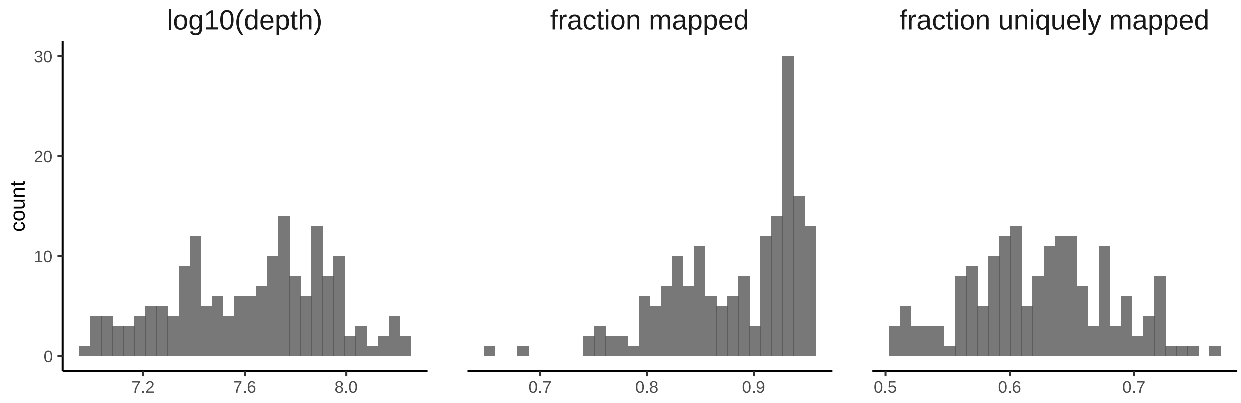

Fig. S7. Mapping statistics from STAR

Distributions of sample mapping statistics from *STAR* [4], including *log*$_{10}$ of the sample read depth (left), fraction of reads that map to the genome (center), and the fraction of uniquely mapping reads (right).

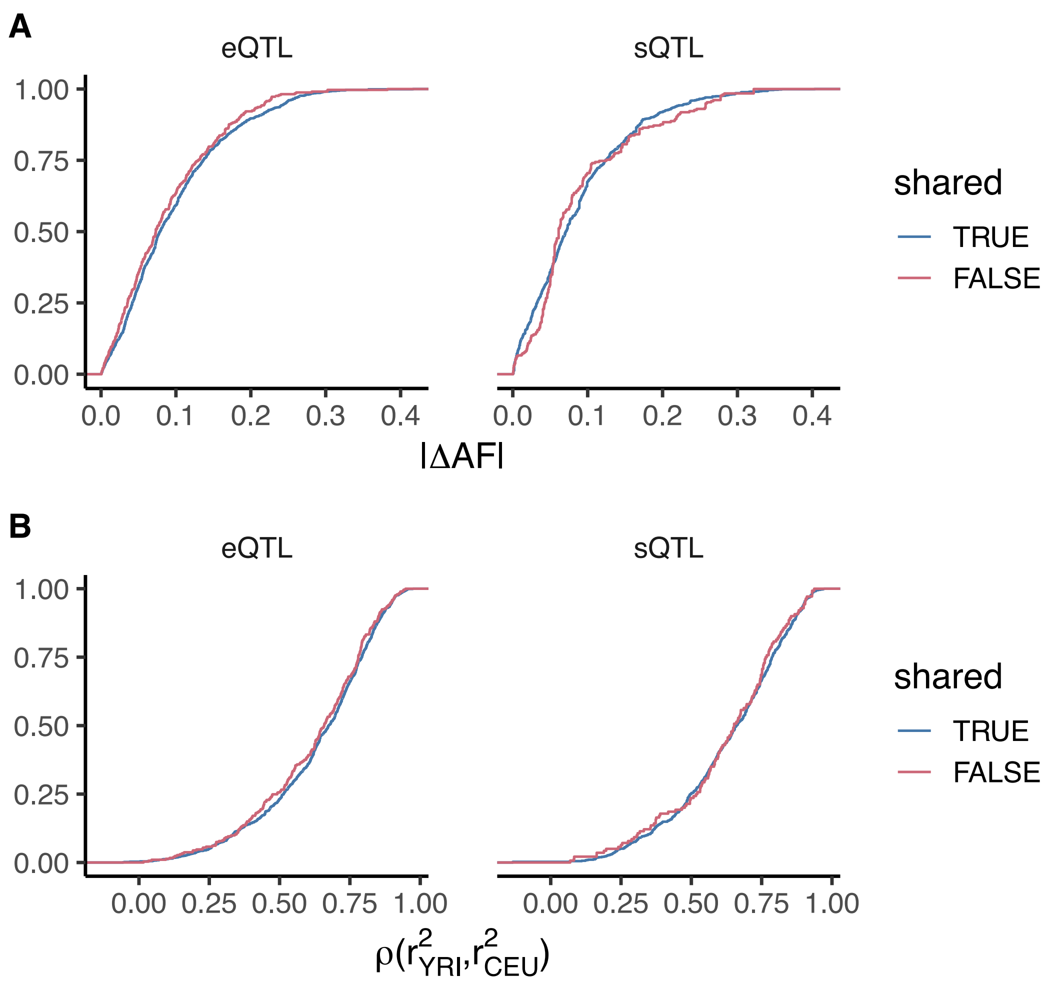

Fig. S8. Frequency and LD differences between African samples and 1000 Genomes EUR populations

tQTL signals that do not remain FDR-significant after conditioning on independent GTEx tQTLs are coded as "shared." **A)** The allele frequency difference of top eQTLs (left) and sQTLs (right) between our cohort and 1000 Genomes EUR populations. Independent African eQTLs are not more likely to show strong frequency differences than shared eQTLs. We do find a weak enrichment of larger frequency differences among sQTLs, but there is no systematic enrichment. **B)** The correlation of African or EUR r^2 statistics estimated between the top eSNP or sSNP and all SNPs within 100kb. We do not find that independent African tQTLs are more likely to show weak correlations in LD.

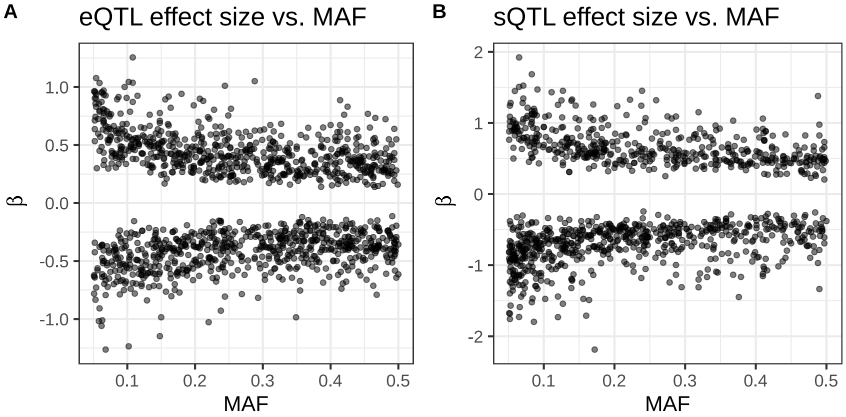

Fig S9. tQTL effect size vs MAF

A) eQTL effect sizes (β) vs minor allele frequency (MAF). **B)** sQTL β vs MAF.

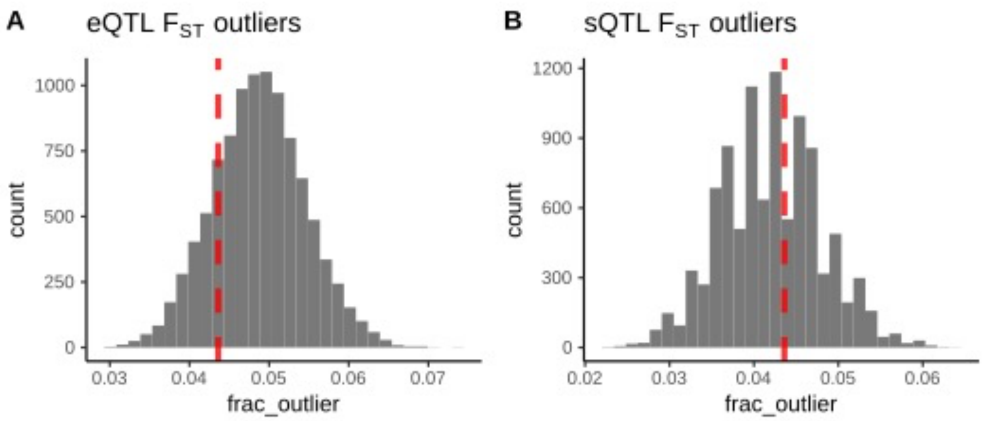

Fig. S10. Fraction of FST outliers among eQTLs and sQTLs compared with matched background

The strongest F_{ST} signal of SNPs in LD with top eQTLs (left) and sQTLs (right) is compared with SNPs matched on MAF and number of SNPs in LD. Plotted are histograms of the fraction of outlier SNP F_{ST} values among 10,000 replicates (gray) and the observed fraction of outlier SNPs among the African tQTLs (red).

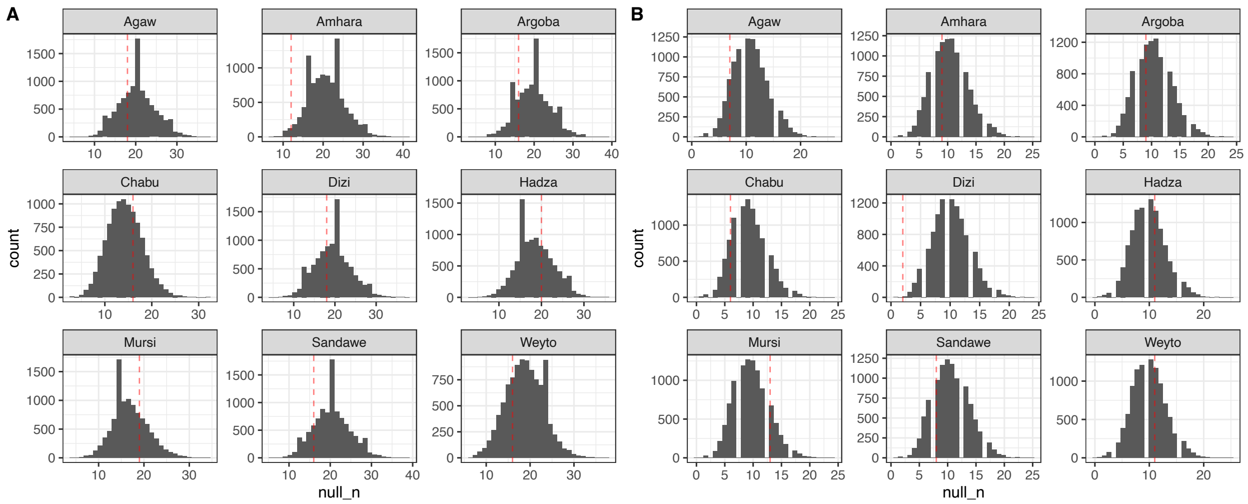

Fig. S11. Population-specific FST outliers

The analyses for Figure S8 were repeated in a population-specific manner. **A)** Results for eQTLs among each population. **B)** Results for sQTLs among each population.

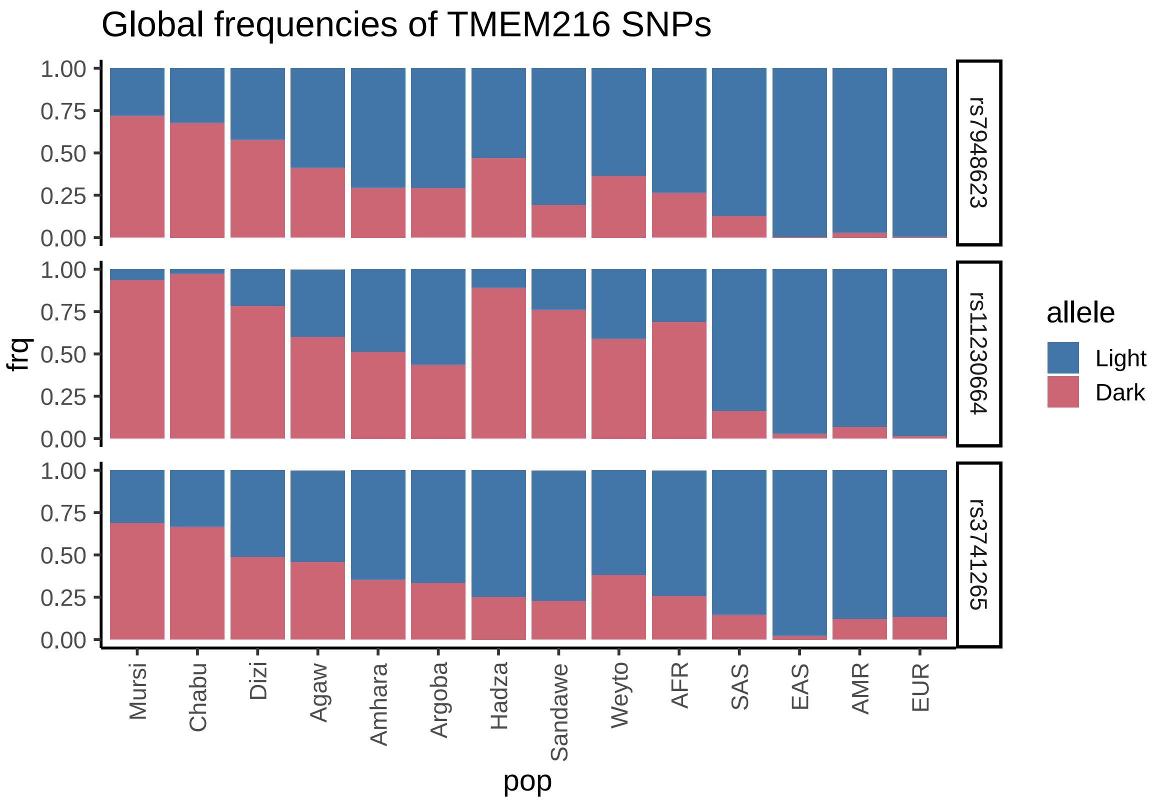

Fig. S12. Global frequencies of SNPs associated with Pigmentation variation and TMEM216 expression and splicing

SNP frequencies for the 9 study populations and 1000 Genome Project super-populations are shown. Alleles are colored by whether they are associated with lighter (blue) or darker (red) skin pigmentation. The Mursi and Chabu show the highest dark allele frequencies at these SNPs among all global populations.

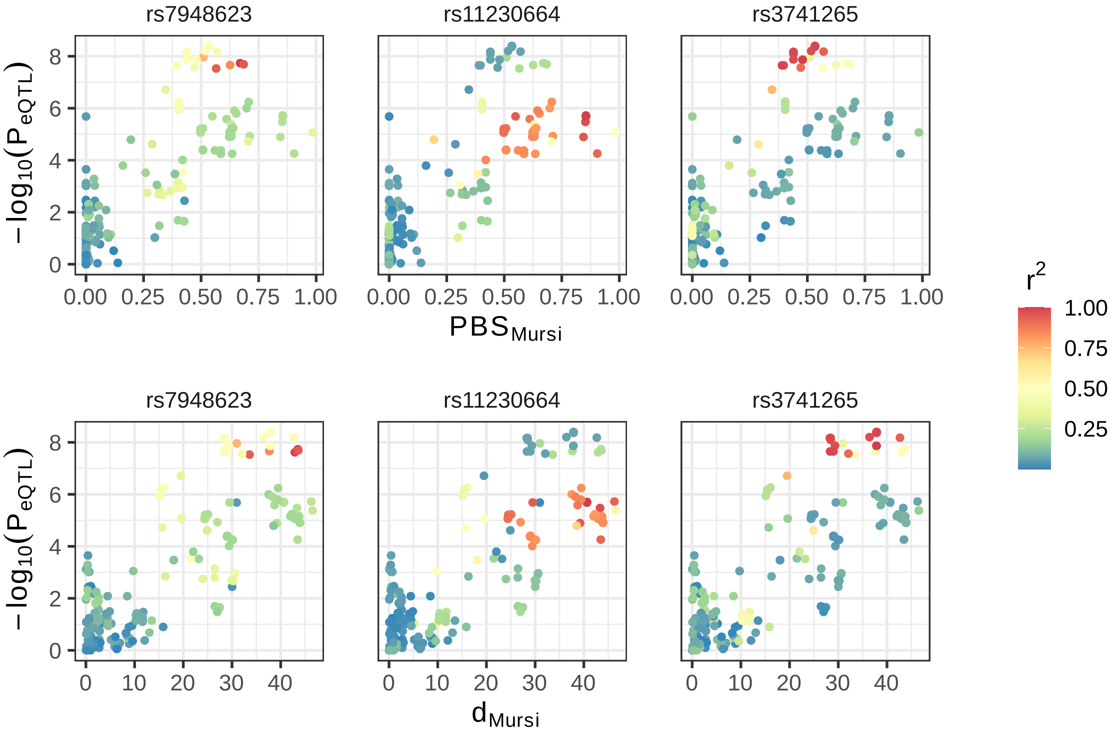

Fig. S13. Colocalization of Mursi *PBS* and *d* with *TMEM216* eQTLs

eQTL p-values (y-axis) are plotted against Mursi *PBS* (top row) and *d* statistics (bottom row). Points are colored by their LD with the top independent pigmentation GWAS SNPs rs7948623 (left column) or rs11230664 (center column), or the sQTL rs3741265 (right column).

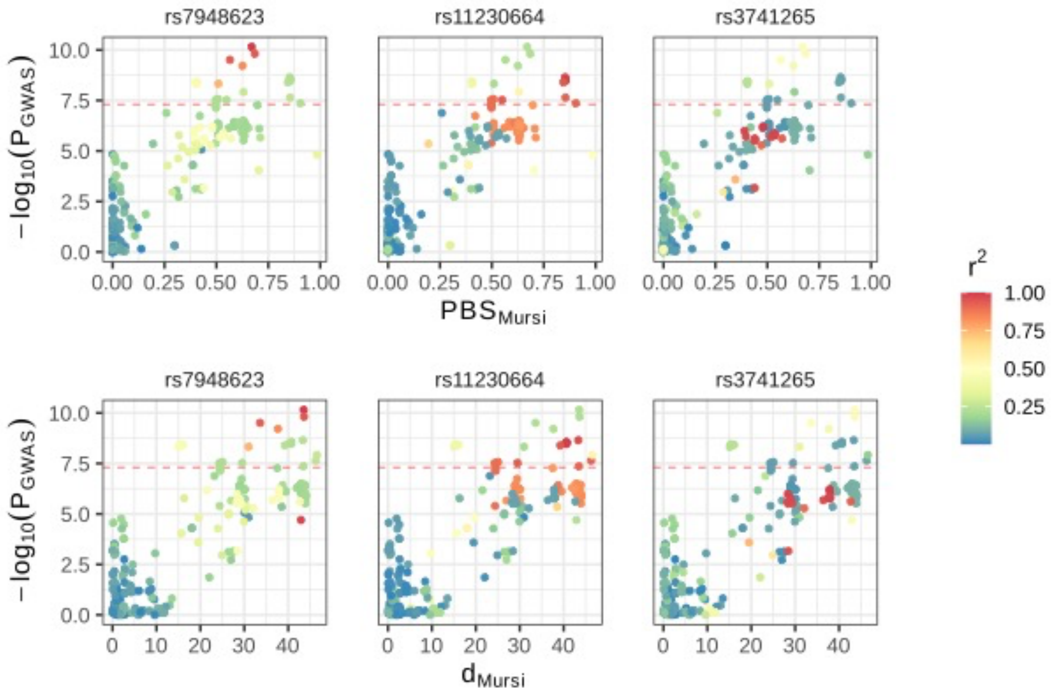

Fig. S14. Colocalization of Mursi PBS and d with pigmentation GWAS

Pigmentation GWAS p-values (y-axis) are plotted against Mursi *PBS* (x-axis, top row) and *d* statistics (x-axis, bottom row). Points are colored by their LD with the top independent pigmentation GWAS SNPs rs7948623 (left column) or rs11230664 (center column), or the sQTL rs3741265 (right column). The red line indicates the GWAS genome-wide significance threshold of $p < 5 \times 10^{-8}$.

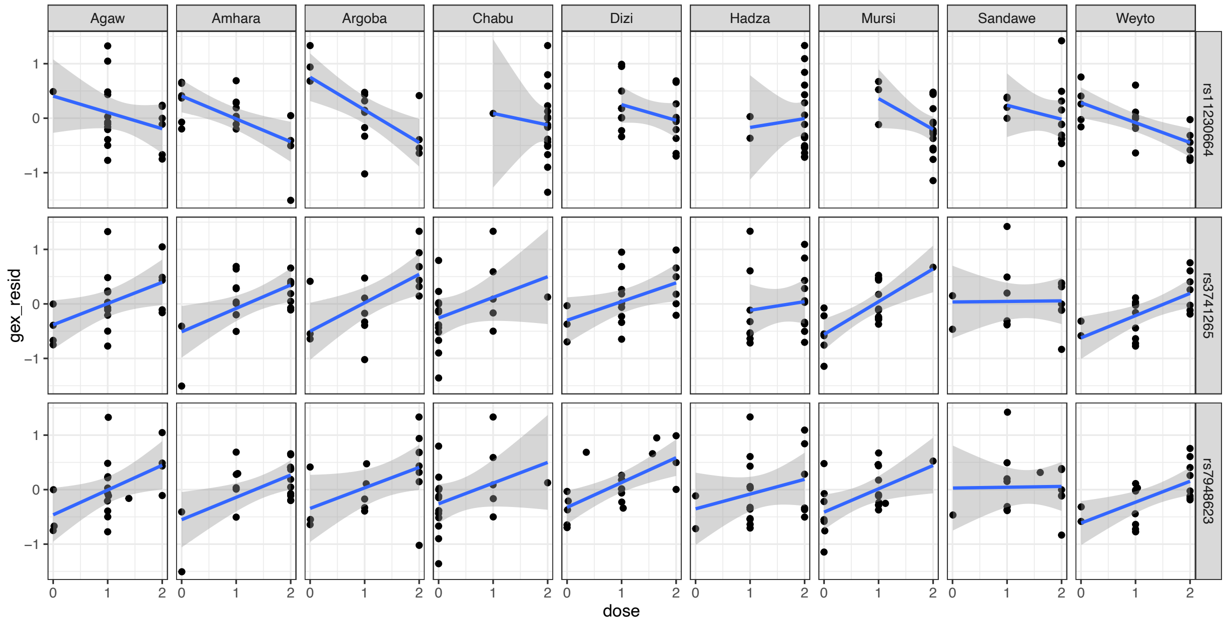

Fig. S15. eQTL associations for *TMEM216* across populations

Normalized expression values for *TMEM216* are plotted against allele dosages for three tag SNPs: rs11230664, and rs3741265, and rs7948623. The consistent effect-size direction across populations suggests our eQTL result at *TMEM216* is robust to population structure.

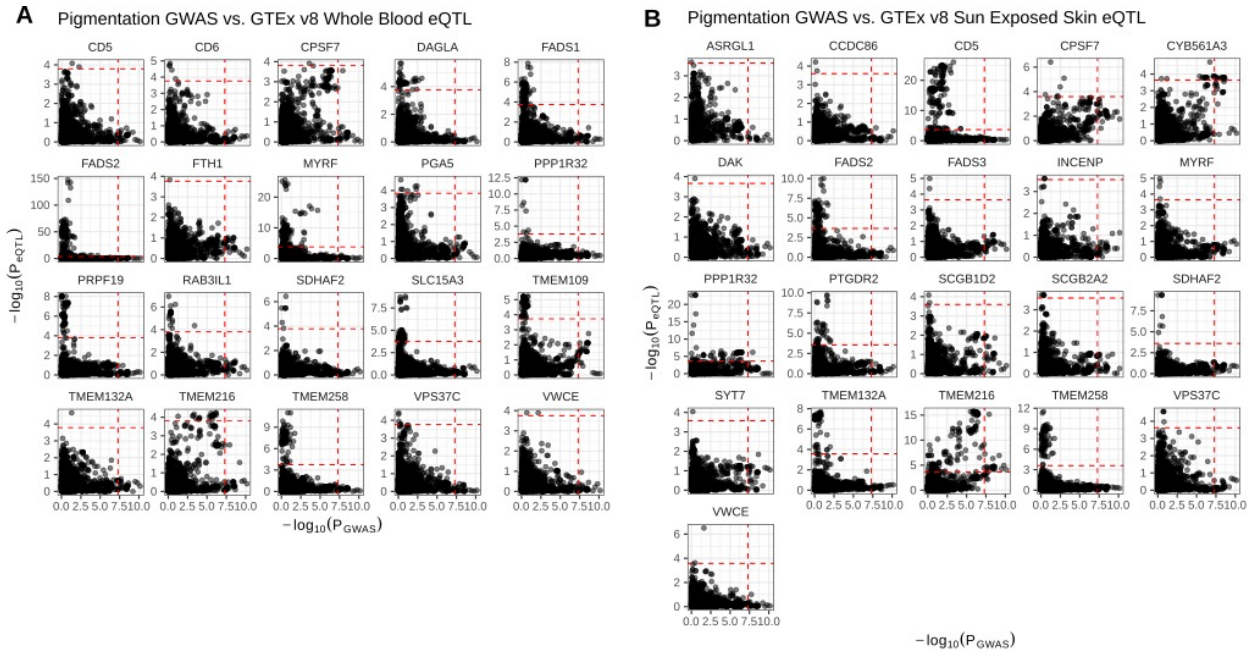

Fig. S16. 'LocusCompare' plots of African Pigmentation GWAS and GTEx v8 eQTLs

Pigmentation GWAS p-values (x-axis) are plotted against GTEx v8 eQTL p-values (y-axis) from either **(A)** Whole Blood or **(B)** Sun Exposed Skin. Vertical dashed lines indicate the threshold for GWAS significance (5 x 10^{-8}) and horizontal dashed lines indicate the FDR < 0.05 significance threshold for each gene.

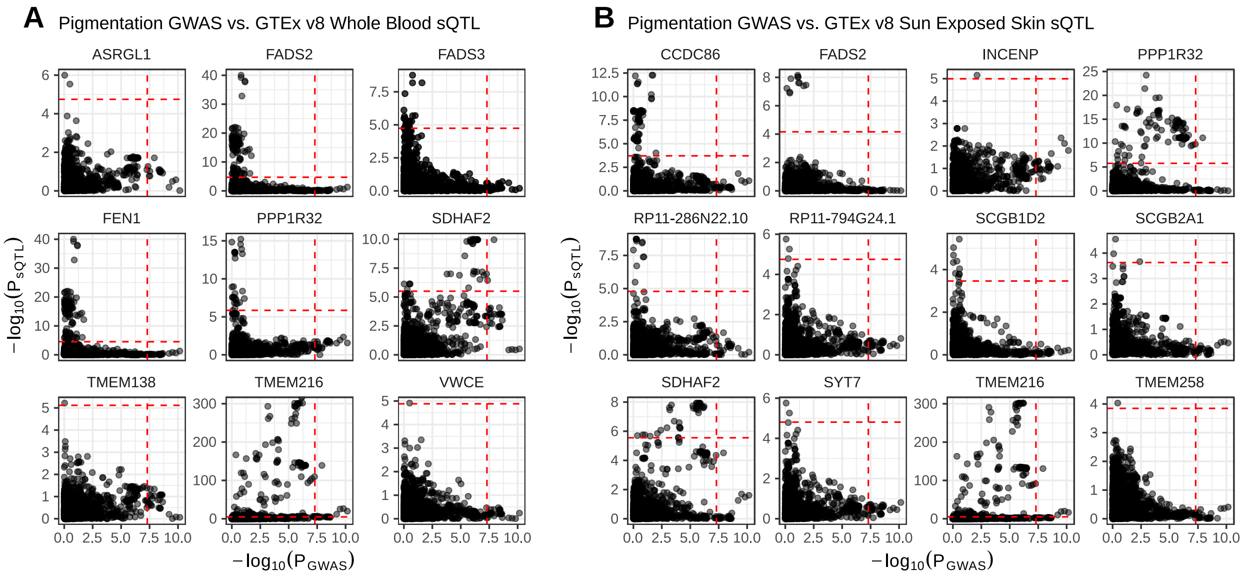

Fig. S17. 'LocusCompare' plots of African Pigmentation GWAS and GTEx v8 sQTLs

Pigmentation GWAS p-values (x-axis) are plotted against GTEx v8 sQTL p-values (y-axis) from either **(A)** Whole Blood or **(B)** Sun Exposed Skin. Vertical dashed lines indicate the threshold for GWAS significance (5 x 10^{-8}) and horizontal dashed lines indicate the FDR < 0.05 significance threshold for each gene.

1. Tehranchi A, Hie B, Dacre M, Kaplow I, Pettie K, Combs P, et al. Fine-mapping cis-regulatory variants in diverse human populations. Morris AP, Wittkopp PJ, editors. eLife. 2019 Jan 16;8:e39595.

2. Dunham I, Kundaje A, Aldred SF, Collins PJ, Davis CA, Doyle F, et al. An integrated encyclopedia of DNA elements in the human genome. Nature. 2012 Sep;489(7414):57–74.

3. McLaren W, Gil L, Hunt SE, Riat HS, Ritchie GRS, Thormann A, et al. The Ensembl Variant Effect Predictor. Genome Biology. 2016 Jun 6;17(1):122.

4. Dobin A, Davis CA, Schlesinger F, Drenkow J, Zaleski C, Jha S, et al. STAR: ultrafast universal RNA-seq aligner. Bioinformatics. 2013 Jan;29(1):15–21.
