# Supplementary material for: The genetic and evolutionary basis of gene expression variation in East Africans
Source: Genome Biol. 2023 Feb 24;24:35. doi: 10.1186/s13059-023-02874-4 (PMC9951478; doi:10.1186/s13059-023-02874-4)
Supplement: Supplementary file 3 — Additional file 3. Reviewhistory. [file 13059_2023_2874_MOESM3_ESM.docx]

**Review history**

**First round of review**

**Reviewer 1**

Kelley et al present an analysis of gene expression and splicing variation in East Africa focusing on whole blood RNA-seq from 162 individuals from Ethiopia and Tanzania. It is inarguably an important area of study to expand analysis to non-European individuals however the study's impact is mostly derived from the focus on East Africa and many of the specific impacts using these data are not yet convincing. I have the following specific comments:
1) The abstract suggests that the genetic architecture of molecular traits "has been less explored" across human populations. Further, the sentence from line 76-78 suggests only recent work here to address a gap. However, there is a history from HapMap (Stranger et al), HGDP, Korean eQTL studies to now Indonesian eQTL to multiple studies on admixed individuals (Price, 2008; GENOA, MESA, others). Further, studies like GEUVADIS and those by Gilad and Pritchard labs have focused on YRI for multiple high impact studies since the earliest days of eQTL studies. I am sure I am missing several here myself but the survey and presentation of the literature in this manuscript is not very complete and the authors should be more specific about what is missing in East Africa.
2) The reference to King and Wilson studying human vs chimp differences as a rationale for studying within population variation is not the best introduction. It could also be misinterpreted that such differences within populations might be as significant as differences between species. A better introduction is that many of human trait association variants appear to be in non-coding regions.
3) I suggest the authors refrain from adjectives like "remarkable" when describing adaptations between humans (line 83).
4) The results on population structure from line 100-111 seem reductive and have no bearing on the analyses presented later in the paper. For example, a "tuber foraging diet" implies what future result in this study?
5) Does window size impact replication (line 143)?
6) Figures 2A/B/C have been presented many times and should be supplemental. The whole section from line 148-160 seems like a QC analysis and could be moved to the supplement.
7) Line 166 replication of sQTLs as eQTLs could also be a technical property of RNA-seq in general. If you have a smaller transcript, the reads mapping to the gene model may in fact not be as accurate as expected (since fewer reads have a chance of being sequenced).
8) Sharing with GTEx suggests very little is novel and the non-replication is also somewhat comparable to the FDR. However, line 189 is a nice analysis suggesting lower MAF could be influential. I would expect that the authors would have looked at more databases than GTEx for replication given the focus on whole blood.
9) Could Line 191 failure to replicate also be due to any lingering population stratification and admixture?
10) Line 200 analyses. There are other sources of whole blood to look at. The GTEx whole blood is known to be non-ideal due to degradation post-mortem.
11) For the SNPs in line 233, how many can be found as eQTLs in the FIVEx eQTL database.
12) Line 273 is expected due to greater diversity. The authors make this seem like a novel finding.
13) How do the authors account for background selection in their Fst analysis? Overall, the signals of selection analysis is very low-pass.
14) Line 339 result, can the authors show that unaccounted population stratification doesn't explain some of these results?
15) Rs7948623 appears to be an eQTL for monocytes too in the FIVEx database.
16) For line 396 analyses showing that DE is present for sensory perception and immunity, the authors should clarify that explained variability is low.
17) The analysis of line 418 should reference studies such as Zaitlen et al, AJHG, 2015 that demonstrate that multi-population analysis refines causal variant detection.
18) Does imputation to only the 1KG impact the quality of the analysis in a population-specific manner?
19) The authors need to make summary statistics available.
20) The authors should consider a new way to represent Figure 5. It is very difficult to interpret and readability is low.
21) Overall, few of the figures are truly novel with respect to what the study highlights and the authors should work to improve this. Figure 1 is genetic data/study design and could be a QC plot. Figure 2 is known. Figure 3a is technical and not the best way to present this result. Figure 4a is hard to interpret. Figure 5A/B are hard to interpret.

**Reviewer 2**

The study by Kelly et al explores the genetic determinants of gene regulation in East Africans by performing expression and slicing QTL mapping in whole blood. The authors find that gene regulation in East Africans is broadly shared with non-Africans, though they also identify novel QTLs. The authors also show that the East African cohort improves the detection of QTLs, and aid in fine-mapping causal loci. Lastly, by integrating their QTL mapping with allele frequency-based selection scores, they find candidate selected genes related to immunity and metabolism. They highlight the TMEM216 locus which shows strong evidence selection in Nilo-Saharan speaking pastoralists that they attribute to pigmentation-related selective pressures. The study represents an important addition to the relatively scarcity of functional studies in non-Europeans. I only have a few suggestions as well as some more general and specific comments that I outline below.

One of my main concerns is the use of an exome-based SNP array to perform QTL mapping. As the authors note in their introduction, "~90% of the genotype-phenotype associations identified in genome-wide association studies (GWAS) cannot be explained by coding variation…" and I wonder how well their SNP array is capturing loci outside genes. I do acknowledge that the authors performed a genome-wide imputation strategy which includes 180 whole-genome sequences from their lab, which I assume includes several East Africans. It would be helpful to know where these individuals are from, specifically, as these could aid in assessing the imputation accuracy. It would also be helpful to show the overall imputation accuracy, as well as the accuracy of their mapped QTLs. That is, it is not clear whether the authors employed a stringent imputation accuracy threshold when conducting their QTL mapping. The authors do mention that they use an r2>0.99 for their population structure analysis (ADMIXTURE, PCA). Was the same cutoff used for their QTL mapping?

My other concern is related to the comparison of mapped loci between the East African cohort and European cohort composed of ~160 individuals. The authors mentioned in their method that "… tQTL mapping was repeated across a range of PEER factors …. and the number of factors maximizing the number of eQTLs or sQTLs discovered were chosen for downstream analysis". However, it the methods section related to the tQTL mapping in Europeans, the authors mention that they corrected for 15 PEER factors, without testing the other sets. I wonder whether this difference could have biased their conclusion regarding the larger number of QTLs in the East African cohort compared to the European one. The authors should also clarify why they used the software GEMMA for the mapping of QTLs in the East Africans, and fastQTL for the Europeans.

Another concern I have is related to some of their statements regarding statistical significance. For example, when exploring the potential factors that may cause the evidence of independent signals in the East African cohorts (MAF and LD), no statistical test is mentioned.

Regarding their enrichment analysis of selection signals at mapped tQTLs, I wonder whether it would also be interesting to test for enrichment in each population separately. That is, showing in their Figure S8 enrichment analysis based on FST computed for each population, separately.

I also noticed that some of the descriptions of their main figures are incorrect. For example, when describing Figure 2, the authors point to panel B when talking about their enrichment analysis. This was the same case for other main figures.

**Authors’ response to reviewers**

Response to Reviewers

We thank the reviewers for their helpful suggestions and have tried to address them as detailed below.

Reviewer 1:

1) 	The abstract suggests that the genetic architecture of molecular traits "has been less explored" across human populations. Further, the sentence from line 76-78 suggests only recent work here to address a gap. However, there is a history from HapMap (Stranger et al), HGDP, Korean eQTL studies to now Indonesian eQTL to multiple studies on admixed individuals (Price, 2008; GENOA, MESA, others). Further, studies like GEUVADIS and those by Gilad and Pritchard labs have focused on YRI for multiple high impact studies since the earliest days of eQTL studies. I am sure I am missing several here myself but the survey and presentation of the literature in this manuscript is not very complete and the authors should be more specific about what is missing in East Africa.

- We appreciate this point and regret neglecting the many groups that have studied genetically diverse populations. We have amended the abstract and lines 76-78 and included relevant citations to better give credit to the history of the field.

2) 	The reference to King and Wilson studying human vs chimp differences as a rationale for studying within population variation is not the best introduction. It could also be misinterpreted ethat such differences within populations might be as significant as differences between species. A better introduction is that many of human trait association variants appear to be in non-coding regions.

- We agree with the reviewer and have removed the section.

3) 	I suggest the authors refrain from adjectives like "remarkable" when describing adaptations between humans (line 83).

- We agree with the reviewer that terms like “remarkable” would best be avoided and have replaced it.

4) 	The results on population structure from line 100-111 seem reductive and have no bearing on the analyses presented later in the paper. For example, a "tuber foraging diet" implies what future result in this study?

- We have removed some of the detail related to the diets of the population used in our study but do believe it is worthwhile to highlight the cultural and genetic diversity of the subjects and their patterns of admixture, which is referenced in the population-specific selection analyses. We have sought to reinforce this in the manuscript.

5) 	Does window size impact replication (line 143)?

- We re-ran eQTL and sQTL mapping using 1mb windows and found 67 new eGenes not discovered using the 100kb window, as well as 108 new introns. However, we had a large reduction in power due to the increased multiple testing burden, with 468 fewer eGenes and 339 fewer sIntrons with 1mb compared with 100kb and believe that, for most of the analyses in our study, the reduction in power outweighs the potential novel findings from using a larger window, though they are certainly worth following up on.

6) 	Figures 2A/B/C have been presented many times and should be supplemental. The whole section from line 148-160 seems like a QC analysis and could be moved to the supplement.

- We agree that these plots have been shown in numerous other papers and serve primarily as QC. We have moved them to the supplement.

7) 	Line 166 replication of sQTLs as eQTLs could also be a technical property of RNA-seq in general. If you have a smaller transcript, the reads mapping to the gene model may in fact not be as accurate as expected (since fewer reads have a chance of being sequenced).

- When binning by gene length deciles we do not find a consistent pattern between gene length and replication as measured by pi1. We have indicated this in the manuscript and have added a supplemental figure.

8) 	Sharing with GTEx suggests very little is novel and the non-replication is also somewhat comparable to the FDR. However, line 189 is a nice analysis suggesting lower MAF could be influential. I would expect that the authors would have looked at more databases than GTEx for replication given the focus on whole blood.

- We extended our replication to data from the eQTLGen consortium, a meta-analysis of blood expression studies, and included the results in the manuscript.

9) 	Could Line 191 failure to replicate also be due to any lingering population stratification and admixture?

- For genome-wide population structure, we believe our LMM approach should sufficiently control for population structure across most genes. We recognize that due to the highly structured nature of our samples some of the tQTLs we detect in Africans but not GTEx may be false positives. When testing those top eQTLs that fail to replicate in GTEx (p > 0.01, 308 total), we find 60 that do not replicate in eQTLGen (p > 0.01, 60 total), suggesting that the majority of the eQTLs that do not replicate between our African samples and GTEx, but which do replicate in eQTLGen (248/308), are likely true positives, and that our false positive rate is in line with our specified FDR of 0.05 (60/1330 = 4.5%). We have added this to our manuscript.

10)	Line 200 analyses. There are other sources of whole blood to look at. The GTEx whole blood is known to be non-ideal due to degradation post-mortem.

- Differential expression across populations is an important and widely studied problem, but our intent here is to explain why some eQTLs don’t replicate. Many of the expression differences are certainly from differences in processing and other technical artifacts, we mean to highlight the specific types of genes that these differences may impact.

11)	For the SNPs in line 233, how many can be found as eQTLs in the FIVEx eQTL database.

- We found that none of those eQTLs or sQTLs are significant in the FIVEx browser, and several are absent.

12)	Line 273 is expected due to greater diversity. The authors make this seem like a novel finding.

- We make a reference to previous work on fine-mapping in diverse populations.

13)	How do the authors account for background selection in their Fst analysis? Overall, the signals of selection analysis is very low-pass.

- We have added a permutation approach to account for background selection, eliminating those selection hits that are in regions of overall high F_ST_. We agree that our approach is simple, but F_ST_ based approaches are common and powerful, and have shown promise in prior research. Because of the nature of our data, tQTLs across common variants, we believe most selection will be on standing variation. We do believe our method of integrating posterior probabilities for tQTLs and scans of selection (rather than more common window-based approaches) is somewhat novel and helps to those genes where the tQTL signal and selection signal in some sense “colocalize.”

14)	Line 339 result, can the authors show that unaccounted population stratification doesn't explain some of these results?

- While we believe that the LMM approach is sufficient to account for population structure, in order to determine if elevated expression of *TMEM216* in populations that also have higher frequencies of pigmentation GWAS variants are due to to a false-positive eQTL result, we performed linear least squares regression between normalized expression values and variant dosages in a population-specific manner. We find consistent effect size directions for all 9 populations for rs7948623 and rs3741265 (p = 3.9 x 10^-3^, two-sided binomial test), and for 8 of the populations for rs11230664 (p = 3.9 x 10^-2^, two-sided binomial test, figure included). Finding a consistent eQTL effect across populations, and for all three unlinked variants, suggests that our eQTL result is robust to population structure.

15)	Rs7948623 appears to be an eQTL for monocytes too in the FIVEx database.

- Thank you, we have added a section mentioning this.

16)	For line 396 analyses showing that DE is present for sensory perception and immunity, the authors should clarify that explained variability is low.

- We have added a section clarifying this point.

17)	The analysis of line 418 should reference studies such as Zaitlen et al, AJHG, 2015 that demonstrate that multi-population analysis refines causal variant detection.

- We have included an appropriate line and reference

18)	Does imputation to only the 1KG impact the quality of the analysis in a population-specific manner?

- Because we are focusing on common variation (MAF > 0.05 in our cohort), it is unlikely that many of the variants we identify would be entirely absent from the 1KG. It would be interesting to know exactly how much inclusion of the 180wgs data improves imputation and the results of our analysis, but that would most likely require re-imputation of our panel and is beyond the scope of this analysis.

19)	The authors need to make summary statistics available.

- We are submitting our summary statistics, raw RNA-seq reads, and genotype data to dbGAP

20)	The authors should consider a new way to represent Figure 5. It is very difficult to interpret and readability is low.

- We have split Figure 5 into two separate figures to improve readability. We have also adjusted many of the gene labels. Plots comparing two different selection statistics have been used previously, but usually for fewer populations. We are open to suggestions for alternative ways of showing our results.

21)   Overall, few of the figures are truly novel with respect to what the study highlights and the authors should work to improve this. Figure 1 is genetic data/study design and could be a QC plot. Figure 2 is known. Figure 3a is technical and not the best way to present this result. Figure 4a is hard to interpret. Figure 5A/B are hard to interpret.

- We recognize that several of our figures are typical of population-genetic studies and eQTL studies, but believe Figure 1 is useful for demonstrating the genetic diversity and population structure of these samples. We agree that the findings in Figure 2 has been demonstrated many times and have moved it to the supplement. There are other ways to present Figure 3A but believe an empirical cumulative distribution plot is effective at demonstrating the enrichment of low MAF tQTL variants in European-ancestry individuals. We have revised Figure 4A and removed the color information, which was not important to our results. To improve the readability of Figure 5, we have separated subplots A and B and worked to improve the readability of Figure 5A (now Figure 4).

Reviewer 2:

Reviewer #2: The study by Kelly et al explores the genetic determinants of gene regulation in East Africans by performing expression and slicing QTL mapping in whole blood. The authors find that gene regulation in East Africans is broadly shared with non-Africans, though they also identify novel QTLs. The authors also show that the East African cohort improves the detection of QTLs, and aid in fine-mapping causal loci. Lastly, by integrating their QTL mapping with allele frequency-based selection scores, they find candidate selected genes related to immunity and metabolism. They highlight the TMEM216 locus which shows strong evidence selection in Nilo-Saharan speaking pastoralists that they attribute to pigmentation-related selective pressures. The study represents an important addition to the relatively scarcity of functional studies in non-Europeans. I only have a few suggestions as well as some more general and specific comments that I outline below.

One of my main concerns is the use of an exome-based SNP array to perform QTL mapping. As the authors note in their introduction, "~90% of the genotype-phenotype associations identified in genome-wide association studies (GWAS) cannot be explained by coding variation…" and I wonder how well their SNP array is capturing loci outside genes. I do acknowledge that the authors performed a genome-wide imputation strategy which includes 180 whole-genome sequences from their lab, which I assume includes several East Africans. It would be helpful to know where these individuals are from, specifically, as these could aid in assessing the imputation accuracy. It would also be helpful to show the overall imputation accuracy, as well as the accuracy of their mapped QTLs. That is, it is not clear whether the authors employed a stringent imputation accuracy threshold when conducting their QTL mapping. The authors do mention that they use an r2>0.99 for their population structure analysis (ADMIXTURE, PCA). Was the same cutoff used for their QTL mapping?

- We realize we mistakenly wrote that we used an exome array in the manuscript. We used the Illumina Omni5 Beadchip array, which provides genome-wide data. Our 180wgs used for imputation includes samples from the Amhara, Dizi, Hadza, Mursi, Chabu, and Sandawe, which has been added to the methods section. We have also created a figure showing the imputation accuracy of eQTLs and sQTLs compared to the whole genome. Across all genomic variants, we find a median imputation accuracy of 0.95. For tQTL mapping we used a cutoff of 0.3, which is referenced in the methods section.

My other concern is related to the comparison of mapped loci between the East African cohort and European cohort composed of ~160 individuals. The authors mentioned in their method that "… tQTL mapping was repeated across a range of PEER factors …. and the number of factors maximizing the number of eQTLs or sQTLs discovered were chosen for downstream analysis". However, it the methods section related to the tQTL mapping in Europeans, the authors mention that they corrected for 15 PEER factors, without testing the other sets. I wonder whether this difference could have biased their conclusion regarding the larger number of QTLs in the East African cohort compared to the European one. The authors should also clarify why they used the software GEMMA for the mapping of QTLs in the East Africans, and fastQTL for the Europeans.

- The number of PEER factors was chosen based on prior GTEx analyses, which optimized the number of PEER factors for sample size counts across tissues. This has been indicated in the manuscript. GEMMA was chosen for eQTL mapping in the African samples due to the high degrees of genetic diversity and population structure, and variable amounts of relatedness, which we found fastQTL was unable to address. fastQTL was used for eQTL mapping in the European-American GTEx subset primarily due to its speed and because 5 genotype PCs was found to be sufficient to account for population structure in the full GTEx cohort, which is larger and more genetically diverse. We therefore argue that 5 genotype PCs will be sufficient for accounting for population structure in the smaller and more genetically similar sample of 162 European Americans used in this study.

Another concern I have is related to some of their statements regarding statistical significance. For example, when exploring the potential factors that may cause the evidence of independent signals in the East African cohorts (MAF and LD), no statistical test is mentioned.

- We have added statistical tests for these sections to the manuscript.

Regarding their enrichment analysis of selection signals at mapped tQTLs, I wonder whether it would also be interesting to test for enrichment in each population separately. That is, showing in their Figure S8 enrichment analysis based on FST computed for each population, separately.

- We have performed enrichment analyses for each population separately and do not find a significant enrichment in any population. We have added a section to the manuscript and supplemental figure.

I also noticed that some of the descriptions of their main figures are incorrect. For example, when describing Figure 2, the authors point to panel B when talking about their enrichment analysis. This was the same case for other main figures.

- Thank you for pointing these errors out, they will be corrected in the re-submitted manuscript.

**Second round of review**

**Reviewer 1**

The authors have sufficiently addressed my comments.

I would recommend some final points for them to consider that can potentially clarify any perceived differences between populations which can be exaggerated by PCA or structure plots.
1) Please consider changing figure 2B axis from B(afr) to B(east africa). This is a small detail but I think worthwhile in main figures to make sure it is clear that this is not representative of all of africa.
2) For 1B, please add PVE to PC1 and PC2.
3) Figure 1C could remove CEU and CHB and focus only on structure within Africa.

**Authors’ response to reviewers**

The authors have sufficiently addressed my comments. I would recommend some final points for them to consider that can potentially clarify any perceived differences between populations which can be exaggerated by PCA or structure plots. 1) Please consider changing figure 2B axis from B(afr) to B(east africa). This is a small detail but I think worthwhile in main figures to make sure it is clear that this is not representative of all of africa. We have made this change to figure 2. 2) For 1B, please add PVE to PC1 and PC2. We have included PVE to PC1 and PC2. 3) Figure 1C could remove CEU and CHB and focus only on structure within Africa. We believe the inclusion of non-Africans (specifically the CEU) is helpful for demonstrating the non-African admixture in East African populations. Figure S1 shows PCs 1-4, which helps to demonstrate higher levels of structure in Africa.
